# Intrahepatic cholangiocarcinoma with clear cell type following laparoscopic curative surgery

**DOI:** 10.1186/s40792-020-01041-2

**Published:** 2020-10-07

**Authors:** Takuya Yamamoto, Tomoyuki Abe, Akihiko Oshita, Shuji Yonehara, Yoshio Katamura, Nozomu Matsumoto, Tsuyoshi Kobayashi, Masahiro Nakahara, Hideki Ohdan, Toshio Noriyuki

**Affiliations:** 1 Department of Gastroenterology, Onomichi General Hospital, Onomichi, Hiroshima Japan; 2 Department of Surgery, Onomichi General Hospital, 1-10-23, Hirahara, Onomichi, Hiroshima 722-8508 Japan; 3 Department of Pathology, Onomichi General Hospital, Onomichi, Hiroshima Japan; 4 Department of Gastroenterological and Transplant Surgery, Graduate School of Biomedical and Health Sciences, Hiroshima University, Hiroshima, Japan

**Keywords:** Clear cell type, Intrahepatic cholangiocarcinoma, Laparoscopic hepatectomy

## Abstract

**Background:**

Intrahepatic cholangiocarcinoma (ICC) is the second most common malignancy of primary liver cancer. Among the several pathological types of ICC, only five cases of the clear cell type have been reported, including the one presented below. Here we report a unique case of clear cell type ICC following laparoscopic hepatectomy.

**Case presentation:**

A 67-year-old woman had a history of hepatitis B virus. Computed tomography revealed a ring-like enhanced mass 35 mm in diameter at segment 7 in the early phase. The enhancement was prolonged to the late phase through the portal phase, while the shape was irregular. Ethoxybenzy magnetic resonance imaging revealed that the tumor had a low signal intensity on T1-weighted imaging and a high signal intensity on T2-weighted imaging. Diffusion-weighted images identified that the tumor had remarkably high signal intensity. Tumor enhancement was not detected throughout the tumor in the hepatocyte phase. Upon ICC diagnosis, a laparoscopic S7 subsegmentectomy was performed. The patient’s postoperative course was uneventful. An immunohistochemical examination revealed that the cells tested positive for cytokeratin 7 (CK7), CK19, and CD56 and negative for CK20, CD10, α-fetoprotein, thyroid transcription factor-1. At 2 years after surgery, the patient remains alive without recurrence.

**Conclusions:**

Here we presented a case of clear cell ICC that was treated by laparoscopic hepatectomy. Immunological analysis, especially by CD56 and several CK markers, is helpful for diagnosing this disease.

## Background

Intrahepatic cholangiocarcinoma (ICC) is the second most common malignancy of primary liver cancer and has a dismal prognosis. Previous reports demonstrated a 5-year overall survival rate of around 40% even after curative surgery [1, 2]. The establishment of a systemic perioperative chemotherapy regimen is currently underway. Recently, the efficacy of neoadjuvant chemotherapy for locally advanced ICC was reported [3]. ICC has several types, including clear cell. Cases of clear cell ICC are limited, as only five have been reported including the one presented here; thus, its imaging characteristics and prognosis remain unclear. Here we report a case of clear cell ICC following laparoscopic hepatectomy.

## Case presentation

**Fig. 1:**
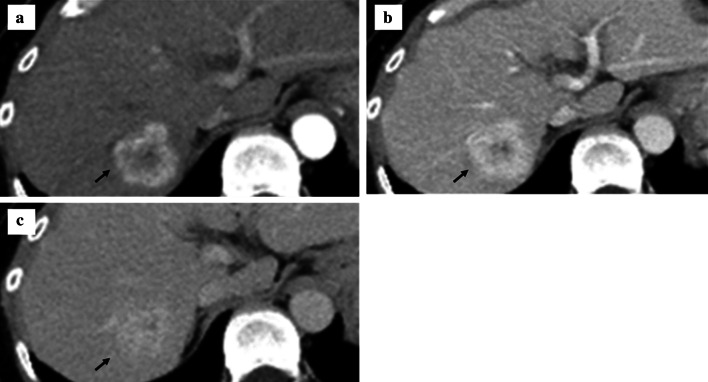
Findings of dynamic abdominal computed tomography. **a** The arterial phase showing a ring-like enhanced mass at segment 7 measuring 35 mm in diameter shaped like an irregular arrow. **b** Portal phase. **c** The enhancement was prolonged to the late phase

**Fig. 2:**
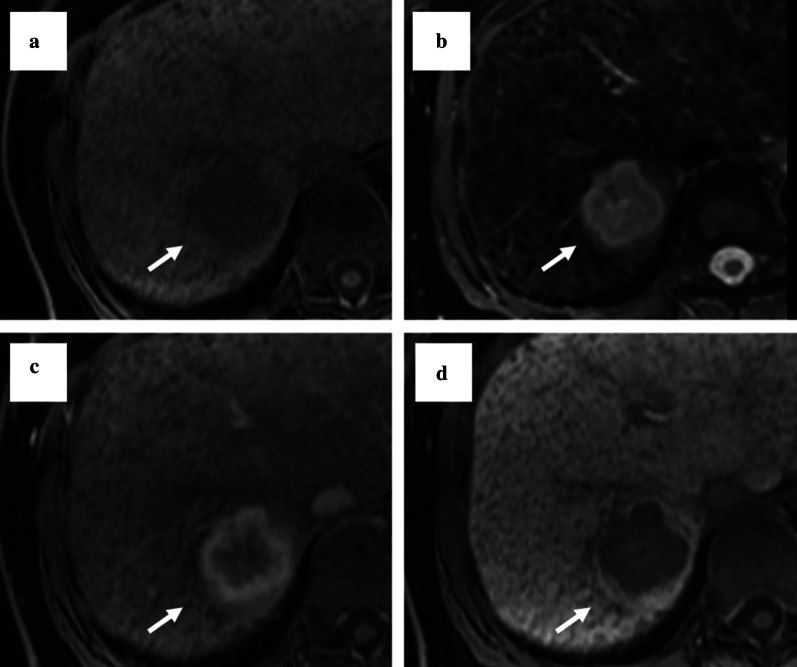
Findings of magnetic resonance imaging (MRI). **a** The tumor shows a low signal intensity on a T1-weighted image. **b** The tumor shows a high signal intensity on a T2-weighted image. **c** At the early phase on ethoxybenzyl magnetic resonance imaging (EOB-MRI), the tumor shows ring-like enhancement. **d** The enhancement is not detected throughout the tumor in the hepatocyte phase on EOB-MRI

**Fig. 3:**
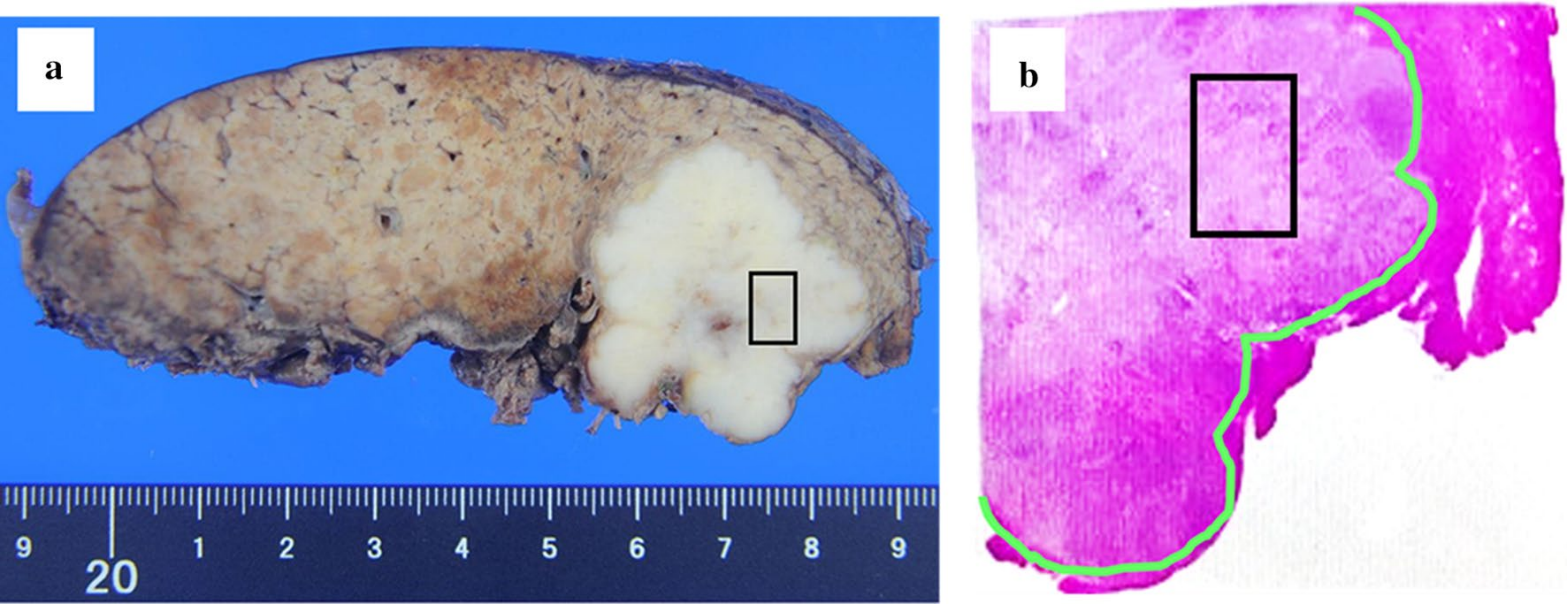
Imaging. **a** Gross appearance of the cut surface showing a solid whitish mass measuring 35 mm × 32 mm × 30 mm with irregular margins. In the area surrounded by the black line on **a** and **b**, clear cells are abundantly clear

**Fig. 4:**
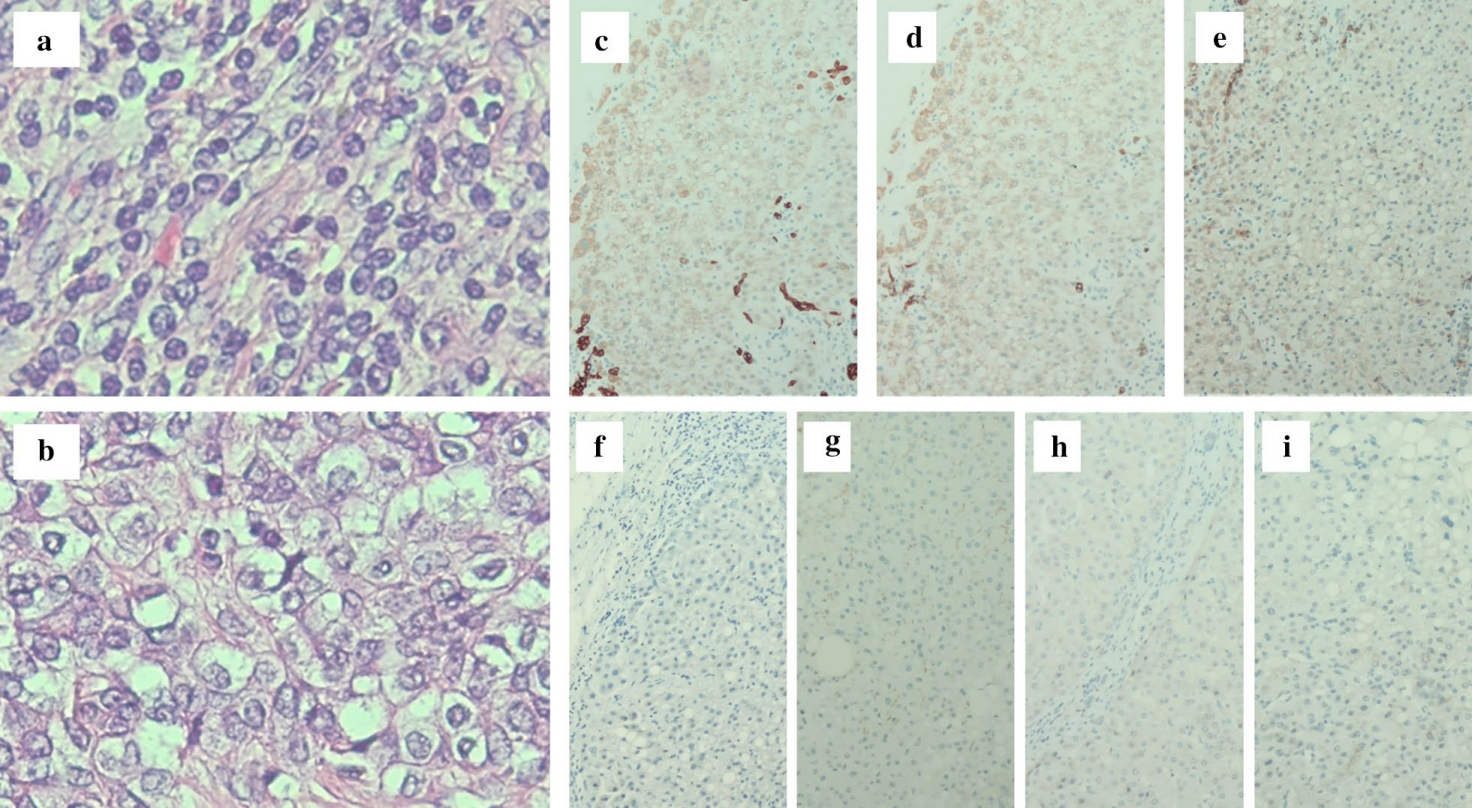
Histopathological findings. **a**, **b** The tumor consists of poorly differentiated adenocarcinoma tissue (**a**) and clear cells with atypical nuclei (**b**) (hematoxylin and eosin stain); **c** CK7 expression is positive; **d** CK19 expression is positive; **e** CD56 expression is positive; **f** CK20 expression is negative; **g** CD10 expression is negative; **h** α-fetoprotein expression is negative; **i** thyroid transcription factor-1 expression is negative

A 67-year-old woman was admitted to our hospital with the chief complaint of high fever. She had a past medical history of hepatitis B virus. Laboratory data showed Child–Pugh liver disease grade A and liver damage grade A. Levels of tumor makers such as carcinoembryonic antigen, carbohydrate antigen 19-9, α-fetoprotein (AFP), and protein induced by vitamin K absence or antagonist-II were within the normal ranges. Dynamic abdominal computed tomography represented a ring-like enhanced mass 35 mm in diameter at segment 7 at the early phase (Fig. 1a). The enhancement was prolonged to the late phase through the portal phase, while the shape was irregular (Fig. 1b, c). Dynamic magnetic resonance imaging revealed that the tumor had a low signal intensity upon T1 weighting (Fig. 2a) and a high signal intensity upon T2 weighting (Fig. 2b). Diffusion-weighted images identified that the tumor had remarkably high signal intensity. The tumors have ring-like enhancement at the early phase (Fig. 2c) that was not detected throughout the tumor at the hepatocyte phase (Fig. 2d). Radiological findings suggested that the tumor was ICC or combined hepatocellular carcinoma (HCC)–cholangiocellular carcinoma for which a laparoscopic S7 subsegmentectomy was performed. On operative finding, the surface of the liver was slightly irregular due to the hepatitis B viral infection. Under intraoperative ultrasonography guidance, the tumor was closed to the right hepatic vein without direct invasion and a laparoscopic S7 subsegmentectomy was completed. The operative time was 528 min, and the intraoperative blood loss was 500 mL. On macroscopic examination, the tumor was solid and whitish with irregular margins (Fig. 3a). The tumor was 35 × 32 × 30 mm in dimension. Microscopically, most of the tumor cells had an enlarged nucleus–cytoplasmic ratio, and they proliferated into funicular or small alveolar structures with stromal tissue consisted of hyaline collagen fiber. The differentiation was poor adenocarcinoma with abundant clear cytoplasm (Fig. 4a, b). An immunohistochemical examination revealed that the cells tested positive for cytokeratin 7 (CK7), CK19, and CD56 (Fig. 4c–i) but negative for CK20, CD10, AFP, and thyroid transcription factor-1 (Fig. 4f–i). Based on these findings, the tumor was diagnosed as a clear cell ICC, T1aN0M0, stage IA according to the American Joint Committee on Cancer/Union for International Cancer Control staging classification 8^th^ edition.

On the 7th postoperative day, she was discharged without complications. Although no adjuvant chemotherapy was administered, her postoperative course was uneventful without a recurrence at 2 year after surgery.

## Conclusions

Histopathologically, ICC develops as various tissues form, such as well-differentiated adenocarcinoma, adenosquamous carcinoma, mucinous carcinoma, mucoepidermoid carcinoma, squamous carcinoma, sarcomatoid carcinoma, and signet-ring cell carcinoma [4, 6]. Among them, the clear cell type is particularly rare. Due to its rarity, its prognosis and radiological findings remain unclear. Nevertheless, radiological characteristic might be sustained intra-tumor staining in the late phases of dynamic CT. Clear cell carcinoma is generally thought to be derived from the ovary and kidney. Some cancers show a clear cell type, such as ICC, HCC, gallbladder carcinoma, and metastasis of lung or thyroid cancer [4, 6–8]. The incidence of clear cell gallbladder carcinoma is reportedly around 1% [7].

Immunohistochemical analysis would play an important role in distinguishing between clear cell HCC and other types. Positivity for CK7 and the absence of CK20 staining are considered features of cholangiocarcinoma [4, 5]. It has been reported that CD56 positivity relates to the clear cell change [3, 9], while ICC shows negativity for vimentin and CD56 [3, 10]. Glycogen, mucin, and lipids also cause the clear cell change [7, 11, 12]; in this case, glycogen reactivity was shown (Fig. 4). We could distinguish clear cell ICC from clear cell HCC in terms of differences in the reactivities of HepPar-l, AFP, and Hepatocyto [4, 9, 11–14]. Renal cell carcinoma is also a well-known clear cell tumor, and its immunohistochemical features include negativity for vimentin, CD10, and S-100 protein [4, 8–11, 15].

Table 1 shows the cases of clear cell type ICC reported to date. Curative surgery was performed in most cases. Among them, three patients survived without recurrence after receiving curative surgery. Based on this observation, clear cell ICC might have a better prognosis than ordinary ICC. Therefore, aggressive surgical resection of this disease is recommended. To the best of our knowledge, this is the first report of a clear cell ICC case undergoing laparoscopic hepatectomy. Laparoscopic hepatectomy is increasingly used to treat primary liver tumors with reportedly fewer postoperative complications. Ban et al. proposed that degree of difficulty of laparoscopic hepatectomy is related to the maximal tumor diameter, tumor location, and liver function. In this case, regardless of a relatively high difficulty score, laparoscopic hepatectomy was achieved [16]. In the present case, no adjuvant chemotherapy was administered and the patient’s postoperative course was uneventful without recurrence for 12 months. Clear cell ICC is a rare malignancy with no effective perioperative chemotherapy; therefore, careful follow-up is necessary to improve postoperative prognosis.

**Table 1:** Reported cases of clear cell type intrahepatic cholangiocarcinoma

Author	Year	Age/sex	Size(cm)	Treatment	Adjuvant chemotherapy	Immunohistochemical analysis	Prognosis
Tihan T, et al.	1998	72/M	15	Surgery	Not described	N/A	Alive at 30 m
Logani S, et al.	1998	64/F	12	Surgery	Not described	N/A	Not described
Falta EM, et al.	1999	50/M	1.5	Surgery	Not described	N/A	Not described
Fernandes SR, et al.	2017	51/F	9.5	Surgery	+	CK7(+), CK20(−)	Alive at 2 yr
Our case	2020	67/F	3.5	Surgery	−	CK7(+), CK19, CD56(−), CK20, CD10, TTF1	Alive at 2 yr

In conclusion, here we presented a case of clear cell ICC following laparoscopic hepatectomy. Immunological analysis, especially by CD56 and several CK markers, is helpful for diagnosing this disease.

## Data Availability

Data sharing not applicable to this article as no datasets were generated or analyzed during the current study.

## Abbreviations

AFP: α-Fetoprotein
CK: Cytokeratin
EOB-MRI: Ethoxybenzyl magnetic resonance imaging
HCC: Hepatocellular carcinoma
ICC: Intrahepatic cholangiocarcinoma
MRI: Magnetic resonance imaging
PAS: Periodic acid–Schiff
